# Specific inhibition of glutamine synthase involved in the metabolic pathway of amino acids is associated with anti-arthritic effects of sinomenine hydrochloride

**DOI:** 10.3389/fcell.2025.1658089

**Published:** 2025-09-03

**Authors:** Gejing Li, Zhaoli Su, Yuanyuan Tang, Hong Huang, Junlan Zhang, Ye Lin, Qin Zhang, Xiong Cai

**Affiliations:** ^1^ Department of Rheumatology, The First Hospital of Hunan University of Chinese Medicine, Changsha, Hunan, China; ^2^ School of Chinese Medical Sciences and Institute of Innovation and Applied Research in Chinese Medicine, Hunan University of Chinese Medicine, Changsha, Hunan, China

**Keywords:** rheumatoid arthritis, sinomenine hydrochloride, metabolomics, adjuvantinduced arthritis, glutamine synthetase

## Abstract

Sinomenine (SIN) is the key bioactive alkaloid isolated from Sinomenium acutum which has been prescribed commonly in Chinese medicine for managing rheumatic disorders. Despite its clinical relevance, the metabolic mechanisms underlying its therapeutic effects remain insufficiently explored, particularly in relation to amino acid dysregulation in rheumatoid arthritis (RA). The anti-arthritic efficacy of sinomenine hydrochloride (SH) was tested in adjuvant-induced arthritis in rats utilizing clinical scoring and histological analysis. Plasma metabolomics was employed to identify SH-mediated changes in amino acid-related metabolic profiles. Key metabolic pathways and targets were examined using computational docking and surface plasmon resonance (SPR) assay. The interaction of SH and molecular targets was further validated in RA fibroblast-like synoviocytes (RA-FLS). SH at dose of 100 mg/kg significantly alleviated disease progression of AIA, as evidenced by reduced paw edema and inhibited histopathological changes. Metabolomic analyses identified 94 potential plasma biomarkers linked to pathways of valine/leucine/isoleucine biosynthesis, glycine/serine/threonine metabolism, phenylalanine metabolism, and alanine/aspartate/glutamate metabolism. Molecular docking and SPR identified that SH specifically targeted the glutamine synthase (GS/GLUL) (KD = 7.12 μM). Experimental validation confirmed that SH (50–200 μM) significantly inhibited GS activity and GLUL expression and consequently decreased glutamine levels in RA-FLS. In conjunction SH exerts significant anti-arthritic effects, partly by modulating the metabolic profiles of related amino acids via selective inhibition of GS-mediated *de novo* Gln synthesis.

## Graphical Abstract

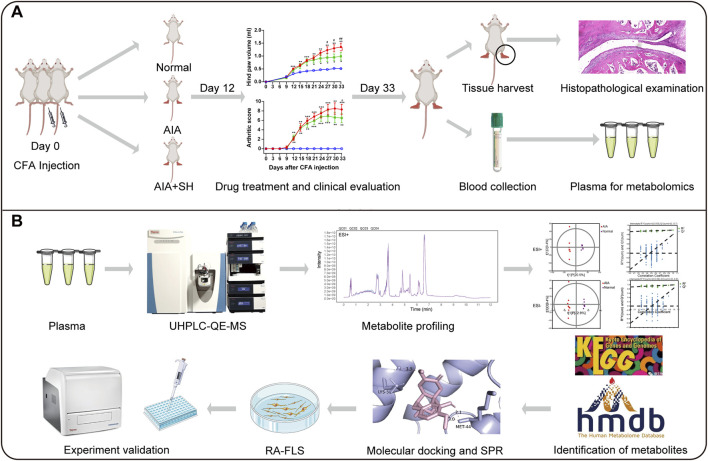

## 1 Introduction

Rheumatoid arthritis (RA) is a systemic autoimmune condition marked by persistent synovial inflammation, abnormal tissue proliferation, and progressive joint degradation involving both cartilage and bone (Di Matteo et al., 2023). Its onset and development are influenced by a complex interplay of genetic predisposition, environmental exposures, and microbial triggers (Scherer et al., 2020). Recent research underscores the metabolic alterations in amino acid pathways among RA patients, suggesting a contributory role in disease progression and a potential utility of these metabolites as biomarkers for disease monitoring and therapeutic response (Panfili et al., 2020). Although conventional therapies—including NSAIDs, corticosteroids, and DMARDs—remain central to RA treatment, their long-term use is constrained by hepatotoxicity and gastrointestinal side effects (Faison et al., 2024). In light of these potential adverse effects, new drug screening is currently underway to develop novel anti-RA agents with enhanced safety profiles.

Sinomenine (SIN), a bioactive alkaloid derived from *Sinomenium acutum* (Thunb) Rehd. et Wils. (Li et al., 2023), has been recognized for its diverse pharmacological activities, including analgesic (Lai et al., 2022), anti-inflammatory (Liu et al., 2018), immunosuppressive (Wang and Li, 2011), and antitumor activities (Zheng et al., 2021). In clinical practice, its hydrochloride form (SH), commonly formulated as ZhenQin Fengtongning (ZQFTN), has long been applied in China for managing RA and related autoimmune disorders (Liu et al., 2018). SH, with fewer side effects, possesses similar efficacy to Western medicines (Huang et al., 2019; Shi et al., 2021). Recent reports have deepened the understanding of its mechanisms in RA therapy. SIN was shown to mitigate inflammation in adjuvant-induced arthritis (AIA) rats by modulating neutrophil activity through suppression of the NF-κB and MAPK axes, as well as inhibiting neutrophil extracellular trap formation (Jiang H. et al., 2023a). Additionally, in collagen-induced arthritis (CIA) models, SIN was observed to alleviate dysbiosis of gut microbiota and influence serum metabolite profiles (Jiang Z. M. et al., 2023b). However, despite the initial elucidation of the mechanism of action, the specific mechanisms underlying SH’s therapeutic effects on RA are not fully understood. Therefore, further investigations are warranted to confirm and gain a comprehensive understanding of the molecular pathways through which SH exerts its effects on RA.

Metabolomics aims to measuring endogenous metabolites to gain a deep understanding of dysregulated metabolic responses to specific diseases (Wishart, 2019). Given the metabolic perturbations in the progression of RA, metabolomics emerges as a potent tool for monitoring disease progression and response to treatment (Xu et al., 2022). Here, UPLC-QE-MS based metabolomics was adopted to characterize metabolic disruptions in AIA rats and to identify metabolic shifts following SH intervention. To further elucidate SH’s therapeutic mechanisms, we integrated molecular docking with experimental assays to pinpoint relevant molecular targets. This combined strategy not only enhances insight into RA-related metabolic remodeling but also accelerates the discovery of candidate targets for drug design.

## 2 Materials and methods

### 2.1 Drugs and reagents

SH (YK-190509, purity ≥ 98%) was provided by the Hunan ZhenQin Pharmaceutical Group (Huaihua, Hunan, China). Trimethyl-d9-amine N-oxide (MD-8020) and nicotinamide-2,4,5,6-[d4] (MD-0041) were purchased from Beijing Manhage Biotechnology (Beijing, China); L-Leucine-d3 (methyl-d3) (IR-21876) from Standard Testing Group (Guangzhou, China); succinic-2,2,3,3-d4 acid (293,075) from Sigma-Aldrich (Shanghai, China); N-benzoyl-d5-glycine (CMS-M0052) from CMASS Scientific (Shanghai, China). All compounds were of ≥98% purity. LC-MS grade methanol and acetonitrile were purchased from CNW Technologies (Dusseldorf, Germany); LC-MS grade ammonium hydroxide from Fisher Chemical (Maharashtra, India); LC-MS grade ammonium acetate and heat-killed *mycobacterium tuberculosis* H37Ra from BD Biosciences (Franklin Lakes, NJ, United States); mineral oil from Sigma-Aldrich (Shanghai, China). RA-derived fibroblast-like synoviocytes (RA-FLS) were procured from Warner Bio Co., Ltd. (Wuhan, Hubei, China); Dulbecco’s modified Eagle’s medium (DMEM), fetal bovine serum (FBS), trypsin, and antibiotics (penicillin-streptomycin) from Gibco (Grand Island, NY, United States). Micro Glutamine Synthetase Assay Kits (BC0915) were brought from Solarbio Science & Technology (Beijing, China); Glutamine Colorimetric Assay Kits (E-BC-K853-M) from Elabscience Biotechnology (Wuhan, Hubei, China). Anti-GLUL (Glutamine synthetase) and anti-GAPDH rat polyclonal antibodies for Western blotting analyses were obtained from Abcam Shanghai Trading Co., Ltd., (Shanghai, China) and Cell Signaling Technology (Shanghai, China); goat anti-rabbit horseradish peroxidase-conjugated secondary antibodies from Elabscience (Wuhan, Hubei, China); phenylmethanesulfonyl fluoride (PMSF) and radioimmunoprecipitation assay (RIPA) lysis buffer from Cell Signaling Technology (Boston, MA, United States); polyvinylidene difluoride (PVDF) membranes from Millipore (Bedford, MA, United States).

### 2.2 Induction of AIA and SH treatment

Twenty-four Sprague-Dawley (SD) rats were randomized into normal control, AIA, and SH groups. AIA, a well-established model for mimicking RA pathology in SD rats, was induced following the protocol outlined by Cai et al. (2006a). In brief, SD rats received a subcutaneous injection of 0.1 mL complete Freund’s adjuvant (CFA) containing 300 μg of heat-killed *Mycobacterium tuberculosis* H37Ra at the tail base. Beginning from day 12 after CFA injection, rats were treated by gavage with either 0.9% sterile normal saline or 100 mg/kg SH which demonstrated significant anti-arthritic effects in our previous study (Zhou et al., 2008).

### 2.3 Animals

Male SD rats (70–90 g) were obtained from Guangdong Vital River Laboratory Animal Technology Co. Ltd. (No. 44829700001151, Foshan, China) and housed at the Laboratory Animal Center of Hunan University of Chinese Medicine (HNUCM; License No. SYXK [Hunan] 2019-0009) under standard conditions with food and water available *ad libitum*. Animal experimentations were authorized by the Institutional Animal Care and Use Committee of HNUCM and conducted following NIH standards for ethical animal use.

### 2.4 Evaluation of arthritis progression

Paw swelling was quantified by examining the volume of both hind paws, with day 0 values set as baseline. Starting on day 9 post-CFA injection, paw edema was assessed every 3 days utilizing a UGO Basile 37,140 plethysmometer (Comerio, Italy), and body weights were recorded concurrently. Arthritis severity was also scored every 3 days utilizing a standardized five-point scale (Cai et al., 2006b). On day 33, all animals were sacrificed via isoflurane overdose. Blood and hind paw tissues were harvested; paws were fixed in 4% paraformaldehyde, decalcified with EDTA, paraffin-embedded, and sectioned for histological analysis as previously reported (Lin et al., 2022; Lin et al., 2024).

### 2.5 Metabolomic analyses

#### 2.5.1 Sample preparation

Following a 24-h fast, 0.7 mL of blood was drawn via the jugular vein into heparinized tubes. Plasma was isolated by centrifugation (3500 × *g*, 15 min, 4 °C). For extraction, 100 μL of plasma was combined with 400 μL of cold acetonitrile/methanol (1:1, v/v) enriched with isotope-labeled internal standards, then vortexed and sonicated for 10 min in an ice bath. The mixture was incubated at −40 °C for 1 h, followed by centrifugation (13,200 × *g*, 15 min, 4 °C). Quality control (QC) samples were generated by pooling equal volumes from each plasma specimen and processed using the same protocol.

#### 2.5.2 UPLC-QE-MS analysis

Plasma metabolite profiling was implemented utilizing a Vanquish^™^ UPLC system coupled with a Q Exactive^™^ HF-X mass spectrometer (Thermo Fisher Scientific, United States). Separation was achieved on an ACQUITY UPLC BEH Amide column (2.1 × 100 mm, 1.7 μm; Waters, United States) with the column oven at 30 °C and autosampler maintained at 4 °C. The mobile phases composed of 25 mmol/L ammonium acetate and 25 mmol/L ammonia hydroxide in water (pH 9.75, phase A) and acetonitrile (phase B). Supplementary Table S1 summarizes the gradient details. The flow rate was set to 0.5 mL/min with a 2 μL injection volume.

Mass spectrometry was implemented in information-dependent acquisition (IDA) mode utilizing Xcalibur software. Full-scan MS spectra were acquired with high consistency. Electrospray ionization (ESI) parameters were optimized as listed in Supplementary Table S2.

#### 2.5.3 Data processing

Raw data were processed utilizing a custom R-based workflow built on the XCMS platform for peak detection, alignment, and integration. Metabolite annotation was performed with an in-house MS2 spectral library, applying a similarity cutoff of 0.3. Processed data were then imported into SIMCA-P (v16.0.2; Sartorius Stedim Data Analytics, Sweden) for principal component analysis (PCA) and orthogonal partial least squares-discriminant analysis (OPLS-DA). Pathway enrichment and topology analyses were implemented via the MetaboAnalyst 4.0 (www.metaboanalyst.ca), referencing the Human Metabolome Database (HMDB) and Kyoto Encyclopedia of Genes and Genomes (KEGG) databases. Pathways with an impact score >0.1 and FDR-adjusted *P* < 0.05 were deemed significantly altered.

### 2.6 Molecular docking simulation

To explore the interaction between SH and its potential targets within key anti-AIA pathways, molecular docking was conducted. The 3D structure of SIN was retrieved from the PubChem website (http://pubchem.ncbi.nlm.nih.gov) and converted to mol2 format employing Openbabel 3.1.1. Protein structures of candidate targets were downloaded from the Research Collaboratory for Structural Bioinformatics (RCSB) Protein Data Bank and preprocessed in AutoDock Vina for docking analysis. Binding interactions were visualized utilizing the PyMOL (v1.5.0.3).

### 2.7 Surface plasmon resonance (SPR) assay

The CM5 sensor chip (Cytiva, Cardiff, Wales, United Kingdom) was modified with recombinant GLUL protein via conventional amine coupling techniques. The interaction kinetics between GLUL and the small molecule SH were evaluated using surface plasmon resonance (SPR) in manual injection mode. A six-point concentration gradient of SH, prepared through 2-fold serial dilution starting from 10 μM, was employed for kinetic analysis. The running buffer was delivered at a flow rate of 30 μL/min, with both the association and dissociation phases lasting 150 s each. Multi-cycle kinetic analysis was performed, yielding sensorgrams that plotted time (s) on the x-axis against response units (RU) on the y-axis, the kinetic parameters were subsequently determined using the BIAcore S200 evaluation software (version 1.1.1, Cytiva, Cardiff, Wales, United Kingdom) through the Steady State Affinity model.

### 2.8 Glutamine synthetase (GS) activity assay

RA-FLS were plated in 10-cm cell culture dishes and maintained in DMEM enriched with 10% FBS and 1% penicillin-streptomycin antibiotic solution. Cell incubation was performed at 37 °C, 5% CO_2_, and 95% humidity utilizing a Forma Steri-Cycle i250 CR CO2 Incubator (Thermo Fisher Scientific, United States). Following a 24-h incubation, the culture medium was refreshed and then cells were treated with varying concentrations of SH (0, 50, 100, and 200 μM). Post-treatment, cells were collected by trypsinization, rinsed twice with phosphate-buffered saline (PBS), and homogenized in ice-cold lysis buffer at a 1:5 (w/v) cell pellet-to-buffer ratio. Enzymatic activity was subsequently determined using a commercial Micro Glutamine Synthetase Assay Kit, with quantitative measurements performed by spectrophotometric analysis at 540 nm wavelength utilizing a microplate reader.

### 2.9 Intracellular glutamine (Gln) detection

RA-FLS (2 × 10^5^ cells/well) were seeded in 6-well plates for 24 h of incubation before treatment with SH at 0, 50, 100, or 200 nM for an additional 24 h. Post-treatment, cells were counted using an automated counter, then lysed in 160 μL of ice-cold 0.9% NaCl. Lysates were subjected to centrifugation (10,000 × g, 15 min, 4 °C), and the supernatant was filtered with the help of 50 kDa molecular weight cut-off ultrafiltration tubes (Amicon Ultra, MilliporeSigma). Gln levels were quantified utilizing a commercial colorimetric assay, with absorbance documented at 450 nm.

### 2.10 Western blotting assay

RA-FLS were plated at a concentration of 3.4 × 10^6^ cells/mL (2 mL per well) in a 6-well plate and cultured for 24 h in DMEM supplemented with 10% FBS and 1% penicillin-streptomycin at 37 °C with 5% CO_2_ in a Thermo Fisher Forma Steri-Cycle i250 CR CO_2_ incubator. Once the cell confluence reached 80%, the cells were subsequently exposed to varying concentrations of SH (0, 50, 100, and 200 μM) for an extended incubation period of 24 h. Subsequently, cells were lysed using RIPA buffer to extract proteins. For immunoblotting, protein samples were separated by 10% SDS-PAGE and transferred onto a PVDF membrane. The membrane was blocked with 5% skim milk for 2 h, followed by an overnight incubation at 4 °C with primary antibodies: GLUL (1:2,500) and GAPDH (1:1,000). After a 1 h incubation with a rabbit secondary antibody, protein levels were detected using the ChemiDoc XRS + system (Bio-Rad, United States). Densitometric quantification was performed using ImageJ 1.8.0 and GraphPad Prism 8 software.

### 2.11 Statistical analyses

Data analyses were completed utilizing the SPSS version 21.0 (IBM, Armonk, NY, United States). The experimental data were summarized as the means ± standard error of means (SEM). Group comparisons were implemented utilizing Student’s two-tailed t-test or one-way analysis of variance (ANOVA), depending on the context. Post hoc comparisons were conducted utilizing Tukey’s test under homogeneity of variance, and Dunnett’s test when variances were unequal. *P* < 0.05 was deemed statistically significant.

## 3 Results

### 3.1 Significance of SH in the progression of AIA

**FIGURE 1:**
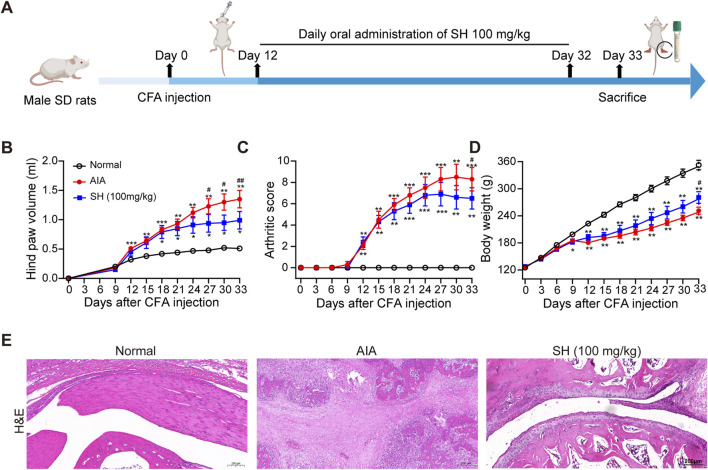
Anti-arthritic evaluation of SH in AIA. **(A)** Experimental workflow, **(B)** hind paw volume, **(C)** arthritic score, **(D)** body weight, and **(E)** H&E staining of normal control, AIA, and SH-treated AIA rats. Data are summarized as the means ± SEM. *P < 0.05, **P < 0.01, and ***P < 0.001 vs. normal control; ^#^ *P* < 0.05 vs. AIA rats.

Anti-arthritic effects of SH were first evaluated in rats with AIA using the experimental protocol schematically outlined in Figure 1A. Compared with normal control rats, a noteworthy increase of 1.35 mL in paw swelling was observed in AIA rats; in contrast, the hind paw volume only increased by 0.99 mL in the SH-treated AIA rats (Figure 1B). In addition, a similar trend was observed for arthritis scores, with the SH group increasing by 6.5 while the AIA group increased by 8.3 (Figure 1C). Additionally, the body weight growth curves suggested that AIA rats exhibited a relatively slower increase during disease progression as compared to SH-treated AIA rats (Figure 1D). Histopathological examination demonstrated that SH markedly inhibited synovial hyperplasia, infiltration of inflammatory cells, and joint space narrowing of AIA rats (Figure 1E). These results indicate the significant inhibition of SH treatment on the paw swelling of AIA rats and the protective effect against inflammation-induced paw swelling.

### 3.2 Plasma metabolite profiling following SH treatment in AIA rats

**FIGURE 2:**
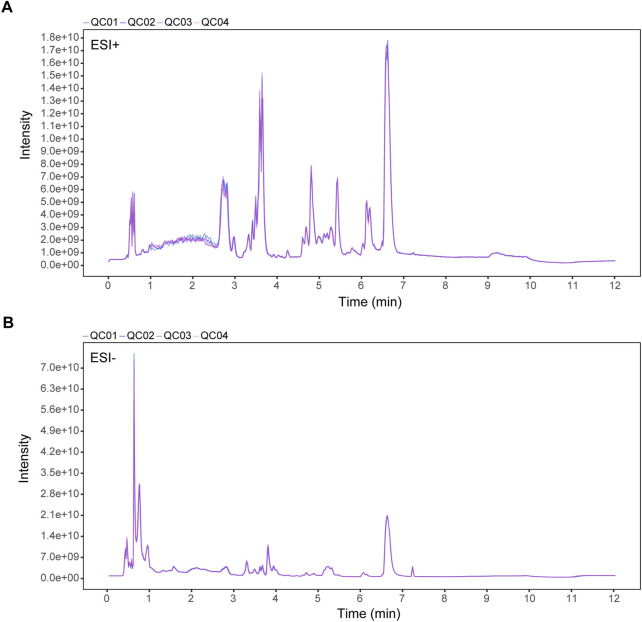
TIC overlaps of QC samples in **(A)** positive (ESI+) and **(B)** negative ion mode (ESI-). X-axis: retention time (min); Y-axis: signal intensity.

To explore SH’s potential mechanisms in AIA, plasma metabolites were profiled in normal, AIA, and SH-treated AIA rats utilizing UPLC-QE-MS in both ESI+ and ESI− modes. The total-ion-current (TIC) chromatograms of QC samples were highly overlapping, indicating the reliability and reproducibility of this system (Figure 2). The PCA-X one-dimensional distribution of QC samples showed that all projections fell within the 2SD interval and gathered scattered points, indicating good precision and stability of the instrument (Supplementary Figure S1).

**FIGURE 3:**
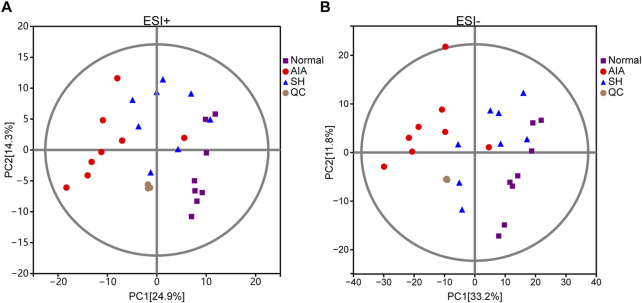
PCA score plots of normal control, AIA and SH-treated AIA, and QC samples in **(A)** positive (ESI+) and **(B)** negative ion mode (ESI-) (n = 8).

The closely clustered PCA score plots of the QC samples also demonstrated the reliability of the experimental results. Owing to the inflammation caused by CFA, the metabolism profiles of AIA exhibited significant alteration as compared with normal control, validating the successful preparation of the AIA model. Following SH treatment, PCA revealed a clear separation between the normal and AIA groups in both ESI+ and ESI- modes, with QC samples tightly clustered, confirming data reliability. SH treatment shifted AIA profiles toward the normal state, particularly in ESI- mode (PC1 = 33.2%), indicating a partial reversal of disease-associated metabolic perturbations (Figure 3).

### 3.3 Differentially regulated metabolites identified in AIA treated with SH

**FIGURE 4:**
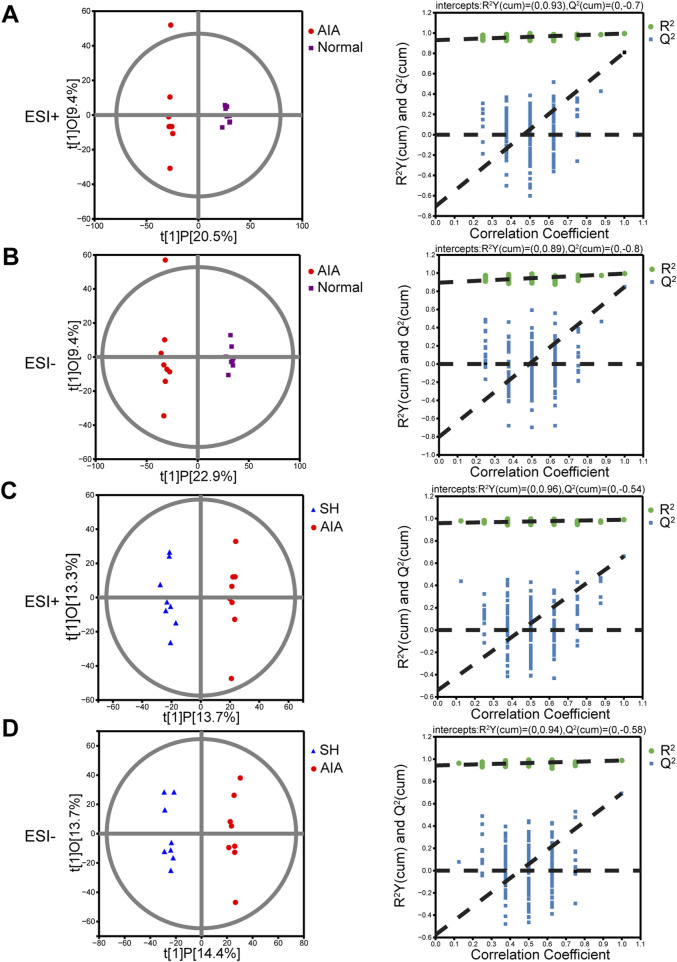
OPLS-DA score plots and permutation tests of normal control, AIA and SH-treated AIA rats, respectively (n = 8). **(A)** Normal control vs. AIA in positive (ESI+) ion mode. **(B)** Normal control vs. AIA in negative (ESI-) ion mode. **(C)** AIA vs. SH in ESI + mode. **(D)** AIA vs. SH in ESI- mode.

The OPLS-DA models demonstrated good performance in identifying the potential metabolites responsible for the metabolic distinction (Madsen et al., 2011). In this study, pairwise comparisons were carried out for establishing OPLS-DA models (AIA vs. normal and AIA vs. SH; Figure 4) to identify the potential metabolites. Seven-fold cross-validation was implemented to assess the OPLS-DA model’s fit (*R* ^2^) and predictive power (Q^2^), with values approaching 1 indicating strong performance (Triba et al., 2015; Carraro et al., 2018). Model robustness was further evaluated through 200 permutation tests. In the comparison between AIA and normal rats, the ESI + model yielded R^2^Y (cum) = 0.93, Q^2^ (cum) = −0.7; for ESI-, values were R^2^Y (cum) = 0.89 and Q^2^ (cum) = −0.8 (Figures 4A,B). Between AIA and SH groups, ESI + mode produced R^2^Y (cum) = 0.96 and Q^2^ (cum) = −0.54, while ESI- showed R^2^Y (cum) = 0.94 and Q^2^ (cum) = −0.58 (Figures 4C,D). These results display reliable interpretation ability of sample categorical information and excellent cross-validated prediction capability, which can be applied for biomarker screening.

**FIGURE 5:**
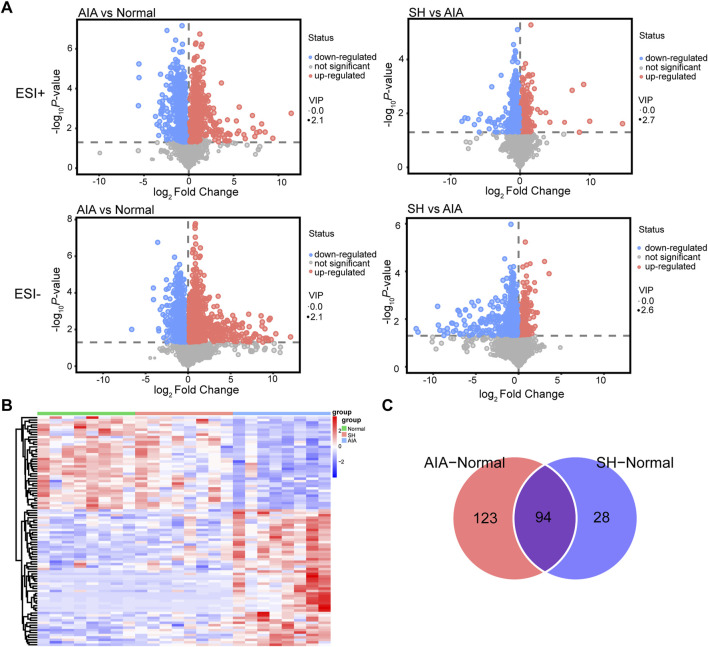
Differential metabolites in SH-treated AIA rats *versus* AIA rats. **(A)** Volcano plot, **(B)** heat maps, and **(C)** Venn diagrams of the potential metabolites.

Metabolites with VIP >1 and *P* < 0.05 in the OPLS-DA model were considered differentially expressed (Zhang et al., 2014), serving as potential biomarkers for distinguishing normal, AIA, and SH groups. These differential metabolites may also shed light on the mechanisms by which SH exerts therapeutic effects on AIA. Then, Volcano plots (Figure 5A) and heat maps (Figure 5B) were used to depict the differences in metabolite levels among the three groups. Two hundred and seventeen differential metabolites in the plasma were found between AIA and normal control, while only 122 differential metabolites were identified between the normal control and SH-treated AIA (Figure 5C). By focusing on the intersection of differential metabolites in these two datasets, 94 endogenous metabolites in plasma were confirmed as potential biomarkers (Figure 5C). Moreover, these metabolites showed a reverse trend in the SH-treated AIA, demonstrating the positive impact of SH treatment on the metabolic perturbation in AIA (Supplementary Table S3; Figure 5B).

### 3.4 Significantly altered metabolic pathways of amino acids with SH treatment

**FIGURE 6:**
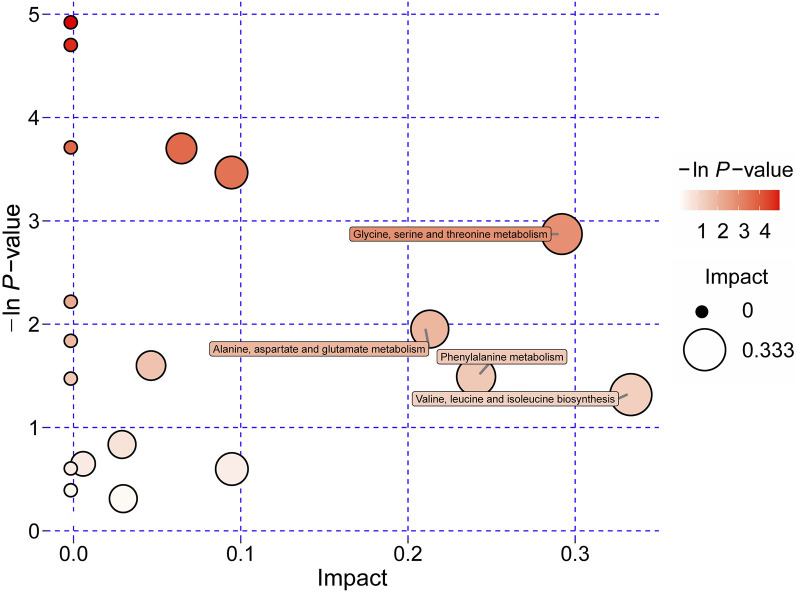
Significantly dysregulated metabolic pathways of amino acids derived from pathway analyses of differentially regulated metabolites in plasma of AIA and SH-treated AIA rats.

**FIGURE 7:**
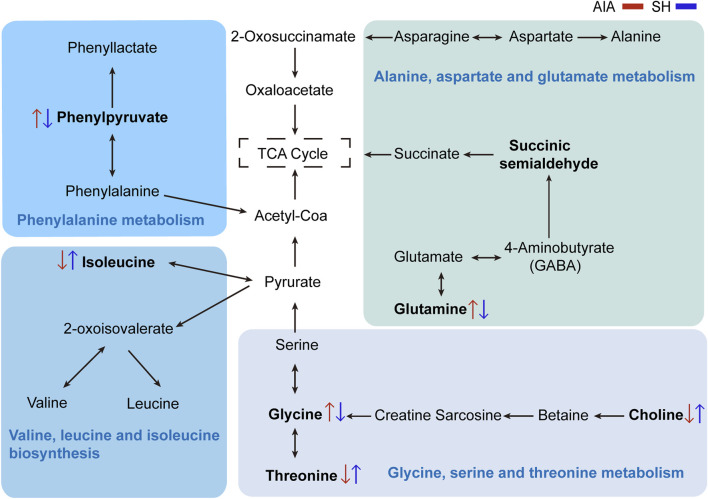
Network visualization of key differential metabolites. Significantly differential metabolites are highlighted in bold. The red and blue arrows represent the changes in these metabolites in AIA and SH-treated AIA rats, respectively.

Integrated pathway enrichment analysis demonstrated significant dysregulation of four amino acid metabolism pathways, i.e., the valine/leucine/isoleucine biosynthesis, the glycine/serine/threonine metabolism, the phenylalanine metabolism, and the alanine/aspartate/glutamic acid metabolism (Supplementary Table S4; Figure 6). Furthermore, by utilizing potential differential metabolites identified from the KEGG database, an interaction network diagram was constructed to delineate the pathophysiological interplay between SH treatment and AIA progression (Figure 7).

### 3.5 Molecular docking and SPR identify GLUL as a direct target of SH

**FIGURE 8:**
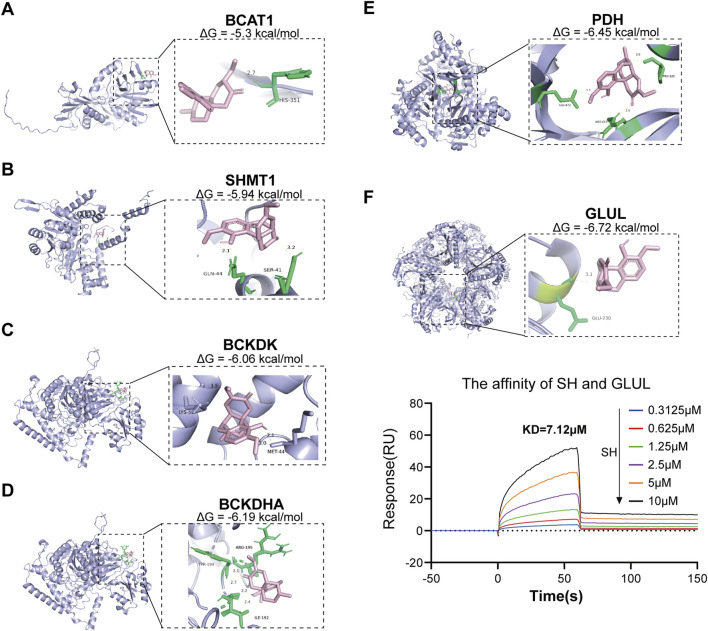
Molecular docking of SIN with six potential targets and the associated binding affinity **(A–F)**, as well as SPR analyses of SH with GLUL **(F)**.

Through integrative cheminformatics analysis, eight key enzymes within the four dysregulated amino acid metabolic axes were identified as putative molecular targets of SH. Table 1 documents these enzymatic targets, including downstream products and expression in RA. Molecular docking simulations demonstrated that the binding affinities between SH and GLUL, PDH, BCKDHA, BCKDK, SHMT1, and BCAT1 were −6.72, −6.54, −6.19, −6.06, −5.94, and −5.3 kcal/mol, respectively (Figure 8). Notably, SH demonstrated superior binding potential to GS. Further SPR revealed 7.12 µM dissociation constant (Kᴅ) of SH to GLUL (Glutamine synthetase [GS]), thereby confirming the high affinity of SH and GLUL (Figure 8).

**TABLE 1:** Potential targets of SIN and their downstream products.

Gene	Protein	Downstream product	Expression in RA	References
*Glul*	Glutamine synthetase	Glutamine	Upregulated	Palmieri et al. (2017)
*Pdh*	[Pyruvate dehydrogenase (acetyl-transferring)] kinase isozyme 1, mitochondrial	Acetyl coenzyme A	Downregulated	Manosalva et al. (2023)
*Bckdha*	2-oxoisovalerate dehydrogenase subunit alpha, mitochondrial	Isovalentyl-coa (from leucine)	Unknown	
		2-methylbutyryl-COA (from isoleucine)		
		Isobutyryl-coa (from valine)		
*Bckdk*	Branched-chain alpha-ketoacid dehydrogenase kinase	The catabolism of KIC produces acetyl-CoA and acetoacetic acid	Unknown	
		KIV is catabolic into succinyl-CoA		
		KVM degradation generates succinyl-CoA and acetyl-CoA		
*Shmt1*	Serine hydroxymethyltransferase	Glycine	Unknown	
*Bcat1*	Branched-chain-amino-acid aminotransferase, cytosolic	2-ketoisohexanoic acid (KIC)	Unknown	
		2-keto-3-methylvaleric acid (KMV)		
		2-ketoisovaleric acid (KIV)		

### 3.6 SH significantly inhibits GS activity and GLUL expression and glutamine levels in RA-FLS

**FIGURE 9:**
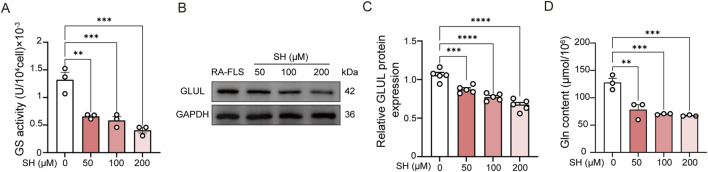
Effects of SH on the GS activity **(A)**, GLUL expression **(B, C)**, and glutamine content in RA-FLS **(D)**. Data are expressed as the means ± SEM. **P < 0.01, ***P < 0.001, and ****P < 0.0001.

The Gln biosynthesized by GS serves as a critical metabolic regulator in the pathological progression of RA-FLS, as evidenced by prior mechanistic studies (Takahashi et al., 2017; Feng et al., 2022). Pharmacological modulation with SH (50, 100, and 200 μM) significantly suppressed GS enzymatic activity (Figure 9A) and GLUL expression (Figures 9B,C), concomitant with a marked depletion of intracellular Gln levels from 127.62 ± 13.35 μmol/10^6^ cells to 67.17 ± 1.16 μmol/10^6^ cells (Figure 9D). This targeted metabolic reprogramming aligns with our global metabolomic signatures (Figure 5B), which revealed coordinated downregulation of glutaminolysis intermediates. The convergence of metabolomic analyses, computational docking, and biochemical assays substantiates SH’s therapeutic mechanism through selective inhibition of GS-mediated *de novo* Gln synthesis.

## 4 Discussion

Various animal models have been established for preclinical evaluation of anti-inflammatory and anti-arthritic agents, with CIA and AIA being among the most widely used due to their clinical relevance and reproducibility (Choudhary et al., 2018). In this study, AIA rats exhibited pronounced paw swelling and erythema, indicative of joint inflammation and structural damage. Notably, the symptoms in AIA rats significantly improved after 18 days of SH treatment, suggesting the function of SH for effectively alleviating the condition of AIA rats.

Rheumatic conditions characterized by inflammatory dysregulation and overproduction of pro-inflammatory factors and cytokines can lead to muscle atrophy and increased energy expenditure (Weyand and Goronzy, 2021). The inflammatory processes in muscle cells then dysregulate the protein degradation pathways and disrupt metabolic processes, resulting in a surge in circulating amino acids (Xu et al., 2022). Furthermore, disorders in amino acid metabolism and abnormal expression of related derivatives can worsen synovial inflammation and hyperplasia (Zhang et al., 2024). In alignment with prior clinical analyses, elevated plasma levels of glutamate, isoleucine, leucine, histidine, and citrulline were previously documented in individuals with RA (Li et al., 2018). These metabolic shifts may reflect impaired protein turnover and a compensatory mechanism to meet increased energy demands and modulate immune function.

In this study, metabolomic analysis identified 94 plasma metabolites altered by SH treatment in AIA rats, most of which were linked to amino acid metabolism. Among them, isoleucine—a key branched-chain amino acid (BCAA)—serves as an energy source for immune cells, and its dysregulation has been linked to oxidative stress and inflammation. Elevated BCAA levels have also been documented in the synovial fluid of RA-affected joints (Dubey et al., 2019). In our results, isoleucine was declined in AIA rats but partially restored following SH intervention. These data indicate that the effects of BCAAs on anti-inflammation are complex and context-dependent (Ahn et al., 2016).

Glycine regulates the production of superoxide and inhibits TNF-α as well as the activation and nuclear translocation of NF-κB. Disrupted glycine metabolism has been implicated in RA progression, while collagen degradation in RA joints further contributes to reduced glycine availability (Aguayo-Cerón et al., 2023). Choline is derived from phosphatidylcholine, which serves as a pivotal role in cell membrane composition. Choline metabolite levels may decline in RA due to the transfer of degraded membrane components from cartilage into synovial fluid (Shet et al., 2012). Threonine, an essential amino acid abundant in immunoglobulins, is critical in cellular stress responses, IL-6 synthesis and secretion, and enhancement of Toll-like receptor activity on the cell surface (Urbaniak et al., 2019) (Supplementary Figure S2). Additionally, phosphorylation and activation of threonine and other amino acids can potentially stimulate the MAPK pathway, and the resultant activation of the MAPK pathway can mediate inflammatory responses and cytokine production that are critical for RA progression (Yang et al., 2018). Herein, plasma levels of glycine, choline, and threonine, which were notably diminished in AIA rats, were reversed after SH treatment due to its regulatory effects on their metabolism.

Phenylalanine, an essential amino acid, is implicated in numerous physiological functions (Ashe et al., 2019). Accumulation of phenylethylamine and phenylpyruvate can influence the gut microbiota, thereby influencing various aspects of health, including intestinal permeability, systemic immunity, energy metabolism, and inflammation (Dodd et al., 2017). Phenylpyruvate, a key intermediate in the alternative metabolic pathway of phenylalanine catabolism, is particularly elevated under such conditions (Supplementary Figure S3). Elevated levels of phenylpyruvic acid result in phenylketonuria and nerve damage (Blau et al., 2010). Previous studies have reported abnormal phenylalanine metabolism in rats (Tang et al., 2021). In this work, we also found the abnormal elevation of phenylpyruvate in AIA rats, which indicated a phenylalanine metabolic disorder in the body. However, the level of phenylpyruvate was reversed after SH treatment.

Gln, the most prevalent amino acid in the body, is vital in supporting immune cells like lymphocytes, neutrophils, and macrophages—key components of host defense. Its anti-inflammatory activity shows linkage with regulation of signaling axes, including NF-κB and other transcriptional regulators (Li et al., 2016). In RA, RA-FLS display abnormal activation of glycolysis and glutaminolysis, contributing to disease progression (Mahdavifard and Sekhavatmand, 2022). The altered glutamate levels observed in AIA rats are consistent with the metabolomic study that focused on RA. These consistent results indicate that glutaminolysis, the process by which Gln is transformed to glutamate, is extremely active in patients with RA. Further mechanistic investigations demonstrated that SH intervention significantly reduced the GS activity in RA-FLS, which was accompanied by a synchronized decline in its downstream metabolite, Gln. This coordinated suppression of the GS activity and Gln bioavailability confirmed that SH exerts therapeutic effects through the modulation of Gln metabolism.

In conclusion, this study elucidated the mechanisms and key targets of SH against AIA, based on changes in endogenous compounds under pathological conditions. SH significantly attenuated joint swelling and inflammation in AIA rats, with 94 differentially expressed plasma metabolites identified. The variety of metabolites suggests that SH has a broad impact on various metabolic pathways and biological processes involved in arthritis. Notably, SH may exert its anti-arthritic effects by influencing valine, leucine, and isoleucine biosynthesis; glycine, serine, and threonine metabolism; phenylalanine metabolism; and alanine, aspartate, and glutamate metabolism. Metabolomics provides valuable information on abnormal metabolites in RA and guides prevention and treatment strategies (Zhang et al., 2024). Targeted metabolomics, specifically focusing on amino acid metabolism, can explore specific metabolic pathways affected by RA and evaluate the potential anti-arthritic effects of SH. Further studies are warranted to clarify the functional roles of these metabolites and validate their relevance in RA therapy.

## Data Availability

All data generated or analyzed during this study are included in this article and its Supplementary Material.

## References

[B1] Aguayo-Cerón K. A. Sánchez-Muñoz F. Gutierrez-Rojas R. A. Acevedo-Villavicencio L. N. Flores-Zarate A. V. Huang F. (2023). Glycine: the smallest anti-inflammatory micronutrient. Int. J. Mol. Sci. 24, 11236. 10.3390/ijms241411236 37510995 PMC10379184

[B2] Ahn J. K. Kim S. Hwang J. Kim J. Kim K. H. Cha H. S. (2016). GC/TOF-MS-based metabolomic profiling in cultured fibroblast-like synoviocytes from rheumatoid arthritis. Jt. Bone Spine 83, 707–713. 10.1016/j.jbspin.2015.11.009 27133762

[B3] Ashe K. Kelso W. Farrand S. Panetta J. Fazio T. De Jong G. (2019). Psychiatric and cognitive aspects of phenylketonuria: the limitations of diet and promise of new treatments. Front. Psychiatry 10, 561. 10.3389/fpsyt.2019.00561 31551819 PMC6748028

[B4] Blau N. van Spronsen F. J. Levy H. L. (2010). Phenylketonuria. Lancet 376, 1417–1427. 10.1016/S0140-6736(10)60961-0 20971365

[B5] Cai X. Wong Y. F. Zhou H. Liu Z. Q. Xie Y. Jiang Z. H. (2006a). Manipulation of the induction of adjuvant arthritis in sprague-dawley rats. Inflamm. Res. 55, 368–377. 10.1007/s00011-006-6026-x 17122958

[B6] Cai X. Wong Y. F. Zhou H. Xie Y. Liu Z. Q. Jiang Z. H. (2006b). The comparative study of sprague–dawley and lewis rats in adjuvant-induced arthritis. Naunyn-Schmiedeberg's Archives Pharmacol. 373, 140–147. 10.1007/s00210-006-0062-5 16703402

[B7] Carraro S. Baraldi E. Giordano G. Pirillo P. Stocchero M. Houben M. (2018). Metabolomic profile of amniotic fluid and wheezing in the first year of life-a healthy birth cohort study. J. Pediatr. 196, 264–269. 10.1016/j.jpeds.2018.01.012 29548683

[B8] Choudhary N. Bhatt L. K. Prabhavalkar K. S. (2018). Experimental animal models for rheumatoid arthritis. Immunopharmacol. Immunotoxicol. 40, 193–200. 10.1080/08923973.2018.1434793 29433367

[B9] Di Matteo A. Bathon J. M. Emery P. (2023). Rheumatoid arthritis. Lancet 402, 2019–2033. 10.1016/S0140-6736(23)01525-8 38240831

[B10] Dodd D. Spitzer M. H. Van Treuren W. Merrill B. D. Hryckowian A. J. Higginbottom S. K. (2017). A gut bacterial pathway metabolizes aromatic amino acids into nine circulating metabolites. Nature 551, 648–652. 10.1038/nature24661 29168502 PMC5850949

[B11] Dubey D. Kumar S. Chaurasia S. Guleria A. Ahmed S. Singh R. (2019). NMR-based serum metabolomics revealed distinctive metabolic patterns in reactive arthritis compared with rheumatoid arthritis. J. Proteome Res. 18, 130–146. 10.1021/acs.jproteome.8b00439 30376345

[B12] Faison M. N. Davis A. M. Trotter K. C. (2024). Disease-modifying drugs for adult-onset rheumatoid arthritis. JAMA 331, 1055–1056. 10.1001/jama.2023.26504 38451547

[B13] Feng X. Li X. Liu N. Hou N. Sun X. Liu Y. (2022). Glutaminolysis and CD4(+) T-cell metabolism in autoimmunity: from pathogenesis to therapy prospects. Front. Immunol. 13, 986847. 10.3389/fimmu.2022.986847 36211442 PMC9537545

[B14] Huang R. Y. Pan H. D. Wu J. Q. Zhou H. Li Z. G. Qiu P. (2019). Comparison of combination therapy with methotrexate and sinomenine or leflunomide for active rheumatoid arthritis: a randomized controlled clinical trial. Phytomedicine 57, 403–410. 10.1016/j.phymed.2018.12.030 30851515

[B15] Jiang H. Lu Q. Xu J. Huo G. Cai Y. Geng S. (2023a). Sinomenine ameliorates adjuvant-induced arthritis by inhibiting the autophagy/NETosis/inflammation axis. Sci. Rep. 13, 3933. 10.1038/s41598-023-30922-3 36894604 PMC9998614

[B16] Jiang Z. M. Zeng S. L. Huang T. Q. Lin Y. Wang F. F. Gao X. J. (2023b). Sinomenine ameliorates rheumatoid arthritis by modulating tryptophan metabolism and activating aryl hydrocarbon receptor via gut microbiota regulation. Sci. Bull. (Beijing) 68, 1540–1555. 10.1016/j.scib.2023.06.027 37422372

[B17] Lai W. D. Wang S. You W. T. Chen S. J. Wen J. J. Yuan C. R. (2022). Sinomenine regulates immune cell subsets: potential neuro-immune intervene for precise treatment of chronic pain. Front. Cell Dev. Biol. 10, 1041006. 10.3389/fcell.2022.1041006 36619869 PMC9813792

[B18] Li Y. Xiao W. Luo W. Zeng C. Deng Z. Ren W. (2016). Alterations of amino acid metabolism in osteoarthritis: its implications for nutrition and health. Amino Acids 48, 907–914. 10.1007/s00726-015-2168-x 26767374

[B19] Li J. Che N. Xu L. Zhang Q. Wang Q. Tan W. (2018). LC-MS-based serum metabolomics reveals a distinctive signature in patients with rheumatoid arthritis. Clin. Rheumatol. 37, 1493–1502. 10.1007/s10067-018-4021-6 29442259

[B20] Li D. Zhong Z. Ko C. N. Tian T. Yang C. (2023). From mundane to classic: Sinomenine as a multi-therapeutic agent. Br. J. Pharmacol. 182, 2159–2180. 10.1111/bph.16267 37846470

[B21] Lin Y. Yi O. Hu M. Hu S. Su Z. Liao J. (2022). Multifunctional nanoparticles of sinomenine hydrochloride for treat-to-target therapy of rheumatoid arthritis via modulation of proinflammatory cytokines. J. Control Release 348, 42–56. 10.1016/j.jconrel.2022.05.016 35569587

[B22] Lin Y. Tang Y. Yi O. Zhu J. Su Z. Li G. (2024). Graphene oxide quantum dots-loaded sinomenine hydrochloride nanocomplexes for effective treatment of rheumatoid arthritis via inducing macrophage repolarization and arresting abnormal proliferation of fibroblast-like synoviocytes. J. Nanobiotechnology 22, 383. 10.1186/s12951-024-02645-8 38951875 PMC11218134

[B23] Liu W. Zhang Y. Zhu W. Ma C. Ruan J. Long H. (2018). Sinomenine inhibits the progression of rheumatoid arthritis by regulating the secretion of inflammatory cytokines and monocyte/macrophage subsets. Front. Immunol. 9, 2228. 10.3389/fimmu.2018.02228 30319663 PMC6168735

[B24] Madsen R. K. Lundstedt T. Gabrielsson J. Sennbro C. J. Alenius G. M. Moritz T. (2011). Diagnostic properties of metabolic perturbations in rheumatoid arthritis. Arthritis Res. Ther. 13, R19. 10.1186/ar3243 21303541 PMC3241363

[B25] Mahdavifard S. Sekhavatmand N. (2022). Glutamine is a superior protector against lead-induced hepatotoxicity in rats via antioxidant, anti-inflammatory, and chelating properties. Biol. Trace Elem. Res. 200, 4726–4732. 10.1007/s12011-021-03046-w 35478087

[B26] Manosalva C. Alarcon P. Quiroga J. Teuber S. Carretta M. D. Bustamante H. (2023). Bovine tumor necrosis factor-alpha Increases IL-6, IL-8, and PGE2 in bovine fibroblast-like synoviocytes by metabolic reprogramming. Sci. Rep. 13, 3257. 10.1038/s41598-023-29851-y 36828912 PMC9958177

[B27] Palmieri E. M. Menga A. Martín-Pérez R. Quinto A. Riera-Domingo C. De Tullio G. (2017). Pharmacologic or genetic targeting of glutamine synthetase skews macrophages toward an M1-like phenotype and inhibits tumor metastasis. Cell Rep. 20, 1654–1666. 10.1016/j.celrep.2017.07.054 28813676 PMC5575233

[B28] Panfili E. Gerli R. Grohmann U. Pallotta M. T. (2020). Amino acid metabolism in rheumatoid arthritis: friend or foe. Biomolecules 10, 1280. 10.3390/biom10091280 32899743 PMC7563518

[B29] Scherer H. U. Häupl T. Burmester G. R. (2020). The etiology of rheumatoid arthritis. J. Autoimmun. 110, 102400. 10.1016/j.jaut.2019.102400 31980337

[B30] Shet K. Siddiqui S. M. Yoshihara H. Kurhanewicz J. Ries M. Li X. (2012). High-resolution magic angle spinning NMR spectroscopy of human osteoarthritic cartilage. NMR Biomed. 25, 538–544. 10.1002/nbm.1769 21850648 PMC3299852

[B31] Shi Y. Pan H. D. Wu J. L. Zou Q. H. Xie X. Y. Li H. G. (2021). The correlation between decreased ornithine level and alleviation of rheumatoid arthritis patients assessed by a randomized, placebo-controlled, double-blind clinical trial of sinomenine. Engineering 16, 93–99. 10.1016/j.eng.2021.04.014

[B32] Takahashi S. Saegusa J. Sendo S. Okano T. Akashi K. Irino Y. (2017). Glutaminase 1 plays a key role in the cell growth of fibroblast-like synoviocytes in rheumatoid arthritis. Arthritis Res. Ther. 19, 76. 10.1186/s13075-017-1283-3 28399896 PMC5387190

[B33] Tang M. Gao X. Geng T. Chen X. Wang J. Shen C. (2021). Metabolomics analysis of the therapeutic effects of qiwei tongbi oral liquid on rheumatoid arthritis in rats. J. Pharm. Biomed. Anal. 202, 114166. 10.1016/j.jpba.2021.114166 34052551

[B34] Triba M. N. Le Moyec L. Amathieu R. Goossens C. Bouchemal N. Nahon P. (2015). PLS/OPLS models in metabolomics: the impact of permutation of dataset rows on the K-fold cross-validation quality parameters. Mol. Biosyst. 11, 13–19. 10.1039/c4mb00414k 25382277

[B35] Urbaniak B. Plewa S. Klupczynska A. Sikorska D. Samborski W. Kokot Z. J. (2019). Serum free amino acid levels in rheumatoid arthritis according to therapy and physical disability. Cytokine 113, 332–339. 10.1016/j.cyto.2018.10.002 30337216

[B36] Wang Q. Li X. K. (2011). Immunosuppressive and anti-inflammatory activities of sinomenine. Int. Immunopharmacol. 11, 373–376. 10.1016/j.intimp.2010.11.018 21109035

[B37] Weyand C. M. Goronzy J. J. (2021). The immunology of rheumatoid arthritis. Nat. Immunol. 22, 10–18. 10.1038/s41590-020-00816-x 33257900 PMC8557973

[B38] Wishart D. S. (2019). Metabolomics for investigating physiological and pathophysiological processes. Physiol. Rev. 99, 1819–1875. 10.1152/physrev.00035.2018 31434538

[B39] Xu L. Chang C. Jiang P. Wei K. Zhang R. Jin Y. (2022). Metabolomics in rheumatoid arthritis: advances and review. Front. Immunol. 13, 961708. 10.3389/fimmu.2022.961708 36032122 PMC9404373

[B40] Yang G. Chang C. C. Yang Y. Yuan L. Xu L. Ho C. T. (2018). Resveratrol alleviates rheumatoid arthritis via reducing ROS and inflammation, inhibiting MAPK signaling pathways, and suppressing angiogenesis. J. Agric. Food Chem. 66, 12953–12960. 10.1021/acs.jafc.8b05047 30511573

[B41] Zhang H. Fu P. Ke B. Wang S. Li M. Han L. (2014). Metabolomic analysis of biochemical changes in the plasma and urine of collagen-induced arthritis in rats after treatment with huang-lian-jie-du-tang. J. Ethnopharmacol. 154, 55–64. 10.1016/j.jep.2014.03.007 24709313

[B42] Zhang X. Yin M. Zhang D. Cao D. Hou X. Xu Z. (2024). Metabolomics reveals disturbed amino acid metabolism during different stages of RA in collagen-induced arthritis mice. Inflammation 47, 1853–1867. 10.1007/s10753-024-02123-1 39212888

[B43] Zheng X. Li W. Xu H. Liu J. Ren L. Yang Y. (2021). Sinomenine ester derivative inhibits glioblastoma by inducing mitochondria-dependent apoptosis and autophagy by PI3K/AKT/mTOR and AMPK/mTOR pathway. Acta Pharm. Sin. B 11, 3465–3480. 10.1016/j.apsb.2021.05.027 34900530 PMC8642618

[B44] Zhou H. Wong Y. F. Wang J. Cai X. Liu L. (2008). Sinomenine ameliorates arthritis via MMPs, TIMPs, and cytokines in rats. Biochem. Biophys. Res. Commun. 376, 352–357. 10.1016/j.bbrc.2008.08.153 18782565

