# Supplementary material for: Specific inhibition of glutamine synthase involved in the metabolic pathway of amino acids is associated with anti-arthritic effects of sinomenine hydrochloride
Source: Front Cell Dev Biol. 2025 Sep 3;13:1658089. doi: 10.3389/fcell.2025.1658089 (PMC12440940; doi:10.3389/fcell.2025.1658089)
Supplement: Supplementary file 1 [file Table1.docx]

***Supplementary Materials***

Supplementary Table 1. Parameters of gradient elution

| Time (min) | Phase A | Phase B |
| --- | --- | --- |
| 0-0.5 | 5% | 95% |
| 0.5-7 | 5%-35% | 95%-65% |
| 7-8 | 35%-60% | 65%B-40% |
| 8-9 | 60% | 40% |
| 9-9.1 | 60%-5% | 40%-95% |
| 9.1-12 | 5% | 95% |

Supplementary Table 2. Parameters of positive and negative ion mode optimization.

| Conditions | | Parameter |
| --- | --- | --- |
| Sheath gas flow rate | | 30 Arb |
| Aux gas flow rate | | 25 Arb |
| Capillary temperature | | 350 °C |
| Collision energy | | 10/30/ 60 in NCE mode |
| Spray Voltage | 3.6 kV in positive, -3.2 kV in negative | |
| Full ms resolution | | 120000 |
| MS/MS resolution | | 7500 |

Supplementary Table 3. Potential biomarkers associated with anti-arthritic effects of sinomenine hydrochloride (SH) on adjuvant-induced arthritis (AIA)

| No | Potential biomarker | Theoretical m/z value | Measured m/z value | ppm | Fold change value | |
| --- | --- | --- | --- | --- | --- | --- |
|  |  |  |  |  | A | S |
| 1 | Choline | 104.1075 | 104.1075 | -0.235 | 0.703 | 1.347 |
| 2 | Acetamidopropanal | 116.0710 | 116.0712 | -2.040 | 0.743 | 1.293 |
| 3 | L-Isoleucine | 132.1022 | 132.1025 | -2.131 | 0.815 | 1.247 |
| 4 | Deoxycytidine | 228.0981 | 228.0984 | -1.376 | 1.475 | 0.788 |
| 5 | Glycerophosphocholine | 258.1105 | 258.1106 | -0.206 | 0.423 | 2.669 |
| 6 | N4-Acetylcytidine | 286.1040 | 286.1039 | 0.368 | 2.129 | 0.548 |
| 7 | Cytosine | 112.0509 | 112.0511 | -1.616 | 1.488 | 0.799 |
| 8 | DL-Tryptophan | 205.0976 | 205.0977 | -0.303 | 0.382 | 2.091 |
| 9 | L-Methionine | 150.0587 | 150.0589 | -1.630 | 0.773 | 1.333 |
| 10 | Glycine | 74.0238 | 74.0242 | -5.844 | 1.058 | 0.944 |
| 11 | Trigonelline | 160.0373 | 160.0374 | -0.824 | 0.197 | 2.664 |
| 12 | L-trans-4-Methyl-2-pyrrolidinecarboxylic acid | 130.0867 | 130.0868 | -1.137 | 0.266 | 3.468 |
| 13 | Metenamine | 141.1138 | 141.1140 | -1.639 | 1.145 | 0.920 |
| 14 | Allysine | 146.0816 | 146.0817 | -0.902 | 1.675 | 0.754 |
| 15 | N-Ethylglycine | 104.0712 | 104.0712 | 0.035 | 1.174 | 0.947 |
| 16 | Citrulline | 176.1034 | 176.1035 | -0.472 | 0.594 | 1.637 |
| 17 | 2-Isopropylphenyl methylcarbamate | 194.1181 | 194.1181 | 0.219 | 1.030 | 1.016 |
| 18 | N, N-Dimethylguanosine | 312.1308 | 312.1308 | -0.119 | 2.232 | 0.513 |
| 19 | Cis-Caffeoyl tartaric acid | 313.0524 | 313.0560 | -11.645 | 10.296 | 0.138 |
| 20 | D-1-Amino-2-pyrrolidinecarboxylic acid | 131.0900 | 131.0821 | 60.558 | 0.303 | 3.220 |
| 21 | N-Acetylornithine | 175.1082 | 175.1083 | -0.806 | 0.763 | 1.711 |
| 22 | 6-Deoxyfagomine | 132.1024 | 132.1025 | -1.021 | 5.703 | 0.185 |
| 23 | 1-(beta-D-Ribofuranosyl)-1,4-dihydronicotinamide | 257.1138 | 257.1137 | 0.309 | 0.590 | 1.965 |
| 24 | N-Lactoyl ethanolamine | 134.0815 | 134.0817 | -1.202 | 1.671 | 0.865 |
| 25 | Arachidyl carnitine | 456.4062 | 456.4053 | 2.024 | 2.190 | 0.621 |
| 26 | PC (24:0/20:5(5Z,8Z,11Z,14Z,17Z) | 892.6812 | 892.6795 | 1.959 | 1.466 | 0.780 |
| 27 | PC (20:2(11Z,14Z)/15:0) | 772.5859 | 772.5856 | 0.329 | 1.267 | 0.818 |
| 28 | PC (24:1(15Z)/20:5(5Z,8Z,11Z,14Z,17Z) | 890.6654 | 890.6639 | 1.684 | 2.315 | 0.589 |
| 29 | PC (24:1(15Z)/22:4(7Z,10Z,13Z,16Z) | 920.7131 | 920.7108 | 2.477 | 1.430 | 0.762 |
| 30 | PC (P-18:0/16:0) | 746.6080 | 746.6064 | 2.153 | 1.672 | 0.892 |
| 31 | PC (22:2(13Z,16Z)/14:0) | 786.6016 | 786.6013 | 0.321 | 1.846 | 0.621 |
| 32 | PC (18:2(9Z,12Z)/14:0) | 730.5395 | 730.5387 | 1.115 | 1.347 | 0.739 |
| 33 | Carbadox | 263.0776 | 263.0780 | -1.693 | 0.307 | 2.932 |
| 34 | Piperine | 286.1515 | 286.1443 | 25.011 | 1.466 | 0.856 |
| 35 | Demethylated antipyrine | 175.0872 | 175.0871 | 0.619 | 0.429 | 1.824 |
| 36 | PC(22:6(4Z,7Z,10Z,13Z,16Z,19Z)/20:4(5Z,8Z,11Z,14Z) | 854.5706 | 854.5700 | 0.740 | 0.435 | 1.576 |
| 37 | (-)-Matairesinol | 359.1512 | 359.1495 | 4.695 | 0.302 | 2.287 |
| 38 | PC (22:5(7Z,10Z,13Z,16Z,19Z)/18:3(6Z,9Z,12Z) | 830.5718 | 830.5700 | 2.154 | 0.242 | 3.484 |
| 39 | PC (22:2(13Z,16Z)/15:0) | 800.6171 | 800.6169 | 0.288 | 1.676 | 0.722 |
| 40 | PC (16:0/14:0) | 706.5409 | 706.5387 | 3.054 | 2.224 | 0.719 |
| 41 | PC (22:6(4Z,7Z,10Z,13Z,16Z,19Z)/20:0) | 862.6328 | 862.6326 | 0.187 | 1.824 | 0.622 |
| 42 | PC (22:6(4Z,7Z,10Z,13Z,16Z,19Z)/20:1(11Z) | 860.6179 | 860.6169 | 1.197 | 1.438 | 0.698 |
| 43 | PC (22:2(13Z,16Z)/20:2(11Z,14Z)) | 866.6645 | 866.6639 | 0.707 | 1.517 | 0.855 |
| 44 | 4-oxo-Retinoic acid | 315.1962 | 315.1960 | 0.755 | 357.296 | 0.006 |
| 45 | all-trans-Retinoic acid | 301.2170 | 301.2168 | 0.738 | 45.894 | 0.053 |
| 46 | Guanidinosuccinic acid | 176.0711 | 176.0671 | 22.992 | 0.465 | 1.701 |
| 47 | 6-Chloro-N-(1-methylethyl)-1,3,5-triazine-2,4-diamine | 188.0710 | 188.0703 | 3.724 | 0.378 | 2.121 |
| 48 | PC (18:0/18:0) | 790.6239 | 790.6326 | -10.944 | 1.633 | 0.658 |
| 49 | N-Methylnicotinamide | 137.0715 | 137.0715 | -0.338 | 2.037 | 0.454 |
| 50 | Ent-15-Kaurene-17,19-dioic acid | 333.2068 | 333.2066 | 0.519 | 240.521 | 0.004 |
| 51 | PC(22:5(4Z,7Z,10Z,13Z,16Z)/20:5(5Z,8Z,11Z,14Z,17Z) | 854.5711 | 854.5700 | 1.263 | 0.434 | 2.283 |
| 52 | Homo-L-arginine | 189.1350 | 189.1352 | -0.797 | 1.700 | 0.085 |
| 53 | Ent-15,16-Epoxy-1(10),13(16),14-halimatrien-19-oic acid | 317.2118 | 317.2117 | 0.300 | 23.789 | 0.085 |
| 54 | 17a-Ethynylestradiol | 297.1858 | 297.1885 | -9.041 | 160.358 | 0.012 |
| 55 | Lyso PC (14:0/0:0) | 468.3087 | 468.3090 | -0.646 | 0.515 | 1.650 |
| 56 | 5-Acetyl-2,3-dihydro-7-methyl-1H-pyrrolizine | 164.1035 | 164.1075 | -24.373 | 1.591 | 0.662 |
| 57 | Lyso PC (20:4(5Z,8Z,11Z,14Z)) | 544.3411 | 544.3403 | 1.482 | 0.207 | 2.825 |
| 58 | Lyso PC (18:1(9Z)) | 522.3552 | 522.3560 | -1.549 | 0.401 | 1.845 |
| 59 | Heptadecanoyl carnitine | 414.3589 | 414.3583 | 1.436 | 1.759 | 0.616 |
| 60 | L-Glutamine | 145.0612 | 145.0613 | -0.917 | 1.738 | 0.988 |
| 61 | LysoPC (16:1(9Z)/0:0) | 494.3250 | 494.3247 | 0.575 | 0.419 | 1.689 |
| 62 | N'-Hydroxysaxitoxin | 316.1375 | 316.1369 | 2.034 | 0.403 | 2.783 |
| 63 | Juvocimene 2 | 299.2014 | 299.2011 | 0.881 | 140.646 | 0.021 |
| 64 | L-Hexanoylcarnitine | 260.1861 | 260.1862 | -0.276 | 1.849 | 0.606 |
| 65 | Alpha-Hydroxyisobutyric acid | 103.0391 | 103.0395 | -3.823 | 3.228 | 0.474 |
| 66 | Thymidine | 301.1048 | 301.1036 | 3.858 | 2.280 | 0.802 |
| 67 | alpha-Tocopherol | 429.3749 | 429.3733 | 3.620 | 1.405 | 0.828 |
| 68 | Pseudouridine | 243.0625 | 243.0617 | 3.196 | 1.098 | 0.897 |
| 69 | L-Threonine | 118.0501 | 118.0504 | -2.547 | 0.563 | 2.001 |
| 70 | Succinic acid semialdehyde | 101.0234 | 101.0239 | -4.840 | 1.479 | 0.609 |
| 71 | 2-Hydroxystearic acid | 299.2595 | 299.2586 | 3.031 | 0.264 | 2.546 |
| 72 | Prostaglandin A2 | 333.2077 | 333.2066 | 3.220 | 10.776 | 0.054 |
| 73 | D-Glutamine | 145.0609 | 145.0613 | -2.714 | 0.520 | 1.514 |
| 74 | LysoPA (18:1(9Z)/0:0) | 435.2522 | 435.2512 | 2.305 | 0.446 | 1.589 |
| 75 | 4-Hydroxycinnamic acid | 163.0394 | 163.0395 | -0.481 | 1.240 | 0.868 |
| 76 | Prostaglandin E2 | 351.2182 | 351.2171 | 3.010 | 14.904 | 0.229 |
| 77 | Phenylpyruvic acid | 163.0394 | 163.0395 | -0.390 | 1.303 | 0.905 |
| 78 | alpha-Ketoisovaleric acid | 115.0391 | 115.0395 | -3.749 | 1.091 | 0.904 |
| 79 | (R)-lipoic acid | 223.0198 | 223.0701 | -225.477 | 0.996 | 1.042 |
| 80 | Cytidine | 242.0783 | 242.0777 | 2.671 | 1.375 | 0.788 |
| 81 | N-p-Coumaroyloctopamine | 298.1148 | 298.1079 | 23.066 | 0.483 | 2.182 |
| 82 | Pyrrolidonecarboxylic acid | 128.0344 | 128.0348 | -3.123 | 0.721 | 1.379 |
| 83 | Jasmonic acid | 209.1183 | 209.1178 | 2.212 | 1.384 | 1.013 |
| 84 | 5-Hydroxy-L-Tryptophan | 221.0925 | 221.0926 | -0.331 | 0.525 | 1.370 |
| 85 | 12-KETE | 317.2127 | 317.2117 | 3.040 | 32.113 | 0.061 |
| 86 | gamma-Aminobutyric acid | 102.0552 | 102.0555 | -3.143 | 2.654 | 0.659 |
| 87 | Carnosic acid | 331.1923 | 331.1909 | 4.092 | 137.312 | 0.021 |
| 88 | 15-Keto-13,14-dihydroprostaglandin A2 | 333.2077 | 333.2066 | 3.160 | 26.911 | 0.057 |
| 89 | 12-HPETE | 335.2234 | 335.2222 | 3.514 | 5.114 | 0.558 |
| 90 | Racemethionine | 148.0431 | 148.04322 | -0.825 | 0.691 | 1.553 |
| 91 | Creatinine | 114.0667 | 114.0667 | -0.024 | 0.969 | 0.919 |
| 92 | Resveratrol | 227.0648 | 227.0708 | -26.558 | 0.434 | 2.057 |
| 93 | L-Proline | 116.0711 | 116.0712 | -0.738 | 0.979 | 1.196 |
| 94 | 15-Keto-prostaglandin E2 | 349.2026 | 349.2015 | 3.236 | 10.133 | 0.151 |

A: Normal group vs. AIA group.

S: AIA group vs. SH group.

Supplementary Table 4. Related metabolic pathways associated with of potential biomarkers of SH treatment in AIA rats

| Name | Total | Hits | Raw *p* | -Ln (*p*) | Impact |
| --- | --- | --- | --- | --- | --- |
| Valine, leucine and isoleucine biosynthesis | 11 | 1 | 0.26762 | 1.3182 | 0.33333 |
| Glycine, serine and threonine metabolism | 32 | 3 | 0.056576 | 2.8722 | 0.29197 |
| Phenylalanine metabolism | 9 | 1 | 0.22481 | 1.4925 | 0.24074 |
| Alanine, aspartate and glutamate metabolism | 24 | 2 | 0.14189 | 1.9527 | 0.21308 |
| Cysteine and methionine metabolism | 28 | 1 | 0.54966 | 0.59846 | 0.09464 |
| Arginine and proline metabolism | 44 | 4 | 0.031175 | 3.4681 | 0.09442 |
| Pyrimidine metabolism | 41 | 4 | 0.024691 | 3.7013 | 0.06454 |
| Glycerophospholipid metabolism | 30 | 2 | 0.202 | 1.5995 | 0.0463 |
| Primary bile acid biosynthesis | 46 | 1 | 0.73269 | 0.31104 | 0.02976 |
| Butanoate metabolism | 20 | 1 | 0.43343 | 0.83603 | 0.02899 |
| Glutathione metabolism | 26 | 1 | 0.52299 | 0.64819 | 0.00573 |
| D-Glutamine and D-glutamate metabolism | 5 | 2 | 0.0071539 | 4.9401 | 0 |
| Aminoacyl-tRNA biosynthesis | 67 | 6 | 0.008903 | 4.7214 | 0 |
| Nitrogen metabolism | 9 | 2 | 0.024 | 3.7297 | 0 |
| Phenylalanine, tyrosine and tryptophan biosynthesis | 4 | 1 | 0.10682 | 2.2366 | 0 |
| Cyanoamino acid metabolism | 6 | 1 | 0.15598 | 1.858 | 0 |
| Methane metabolism | 9 | 1 | 0.22481 | 1.4925 | 0 |
| Porphyrin and chlorophyll metabolism | 27 | 1 | 0.53651 | 0.62267 | 0 |
| Valine, leucine and isoleucine degradation | 38 | 1 | 0.66266 | 0.4115 | 0 |
| Purine metabolism | 68 | 1 | 0.86005 | 0.15077 | 0 |


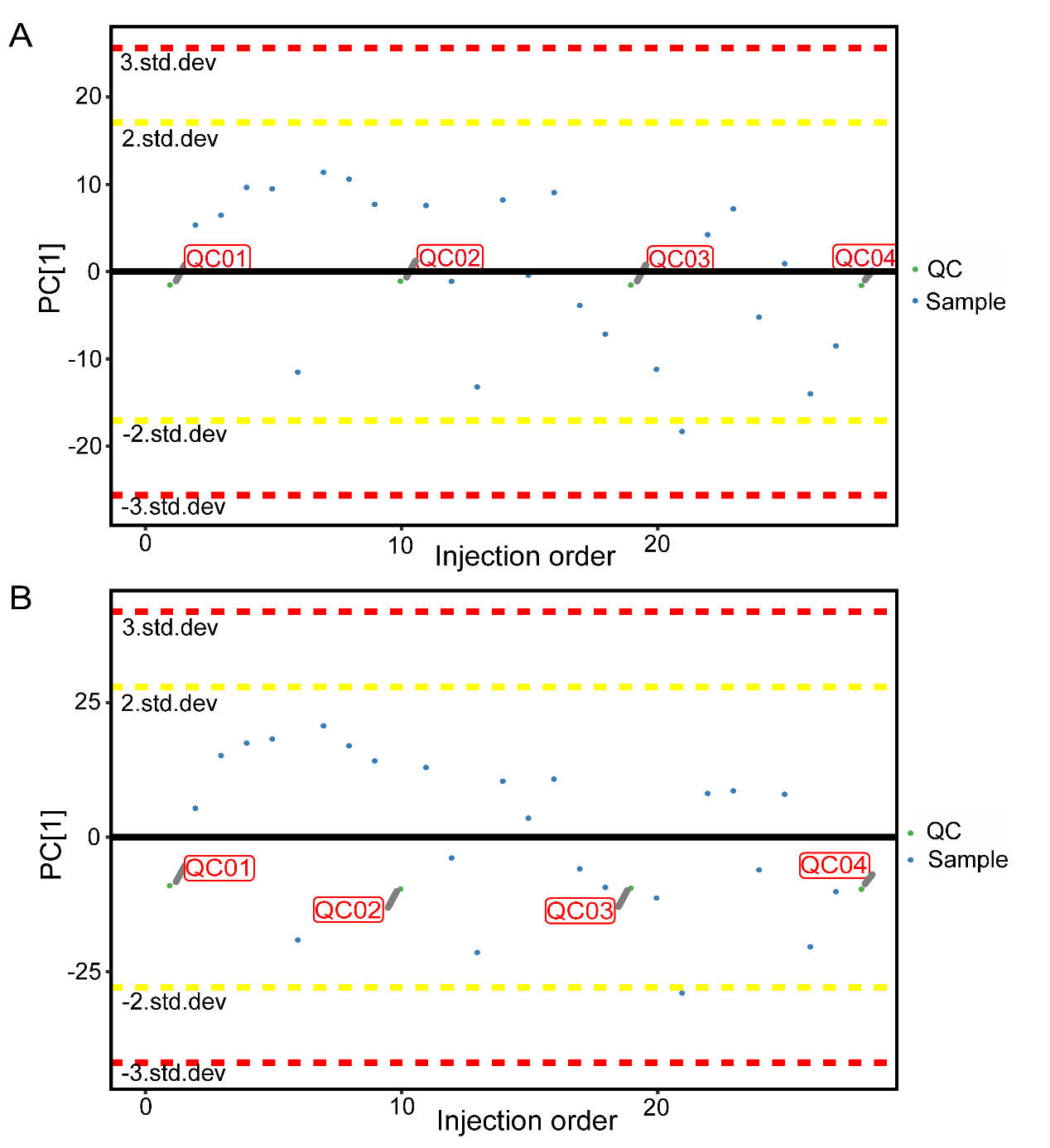


Supplementary Figure 1. PCA-X one-dimensional distribution of QC samples.


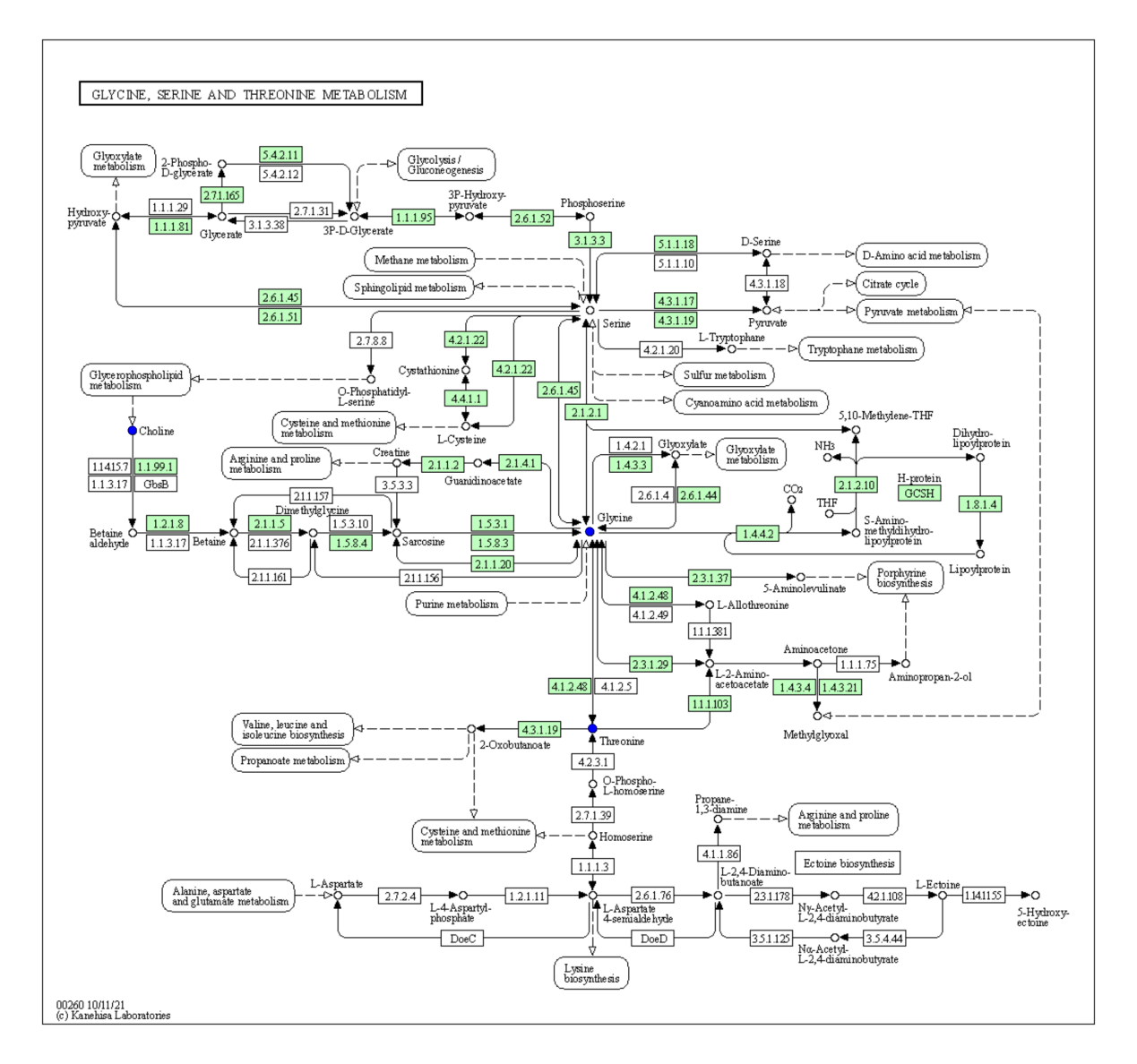


Supplementary Figure 2. KEGG map of glycine, serine and threonine metabolic pathways


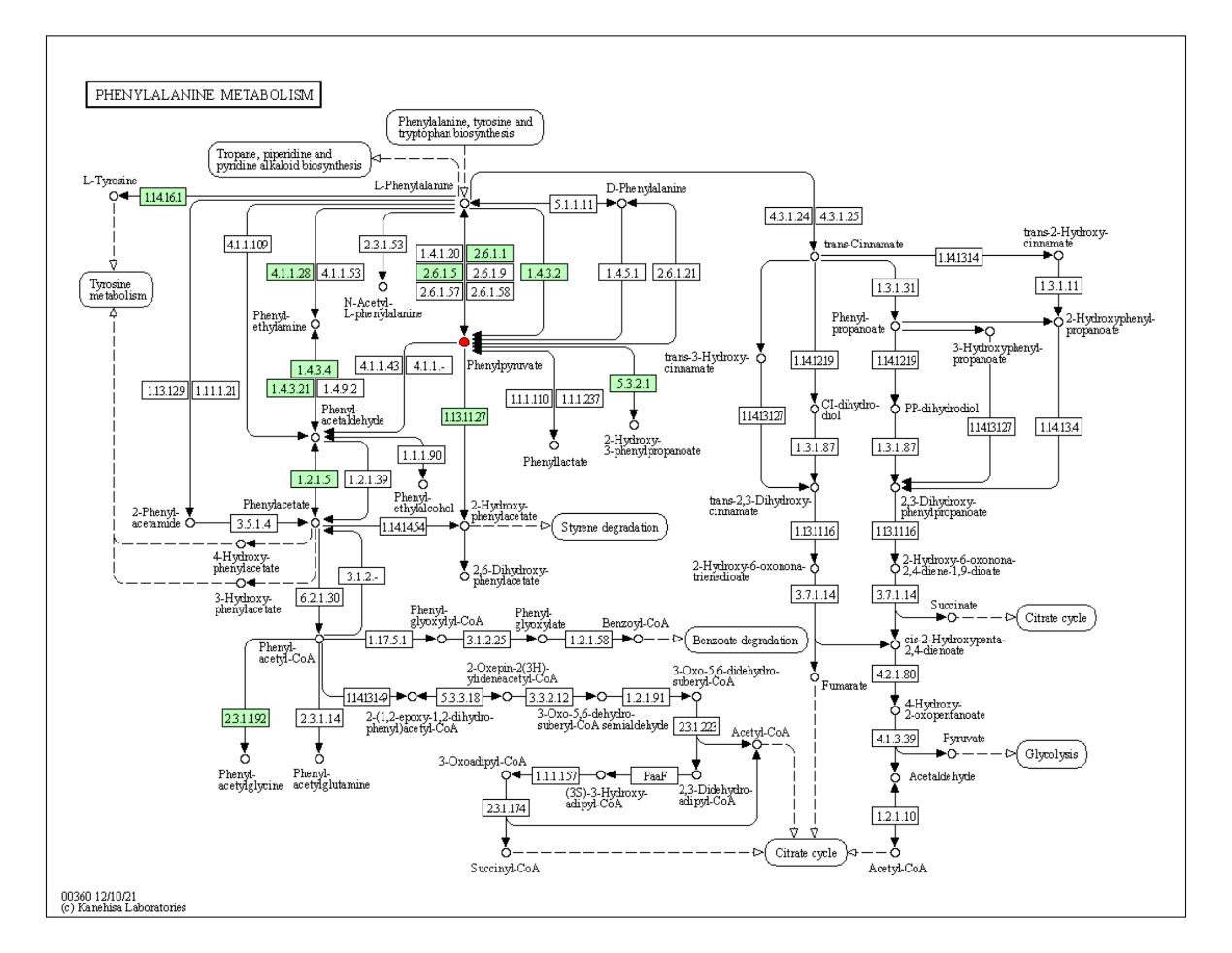


**Supplementary Figure 3**. KEGG diagram of phenylalanine metabolic pathway
